# Seizure‐activated neurons facilitate tau spread in 5XFAD mice

**DOI:** 10.1002/alz.090886

**Published:** 2025-01-03

**Authors:** Aaron J Barbour, Abigail Chavez, Keegan Hoag, Xiaofan Li, Chloe Hassman, Virginia M.‐Y. Lee, Delia M Talos, Frances E Jensen

**Affiliations:** ^1^ University of Pennsylvania, Philadelphia, PA USA

## Abstract

**Background:**

Seizures are highly comorbid with Alzheimer’s disease (AD). We and others have demonstrated worsened pathological and cognitive outcomes in AD patients with seizure history and after seizure induction in AD mouse models. Central to AD progression is the spread of tau along neuronal connections, which can be modelled by intracerebral injection of human AD brain derived tau lysate (AD‐tau), but whether seizures impact the spread of tau is unknown. We hypothesized that seizures would worsen tau spread and that neurons activated during seizures would have increased susceptibility to develop and possibly transmit tau pathology.

**Methods:**

To investigate seizure‐tau interactions, we crossed the 5XFAD mouse model with targeted recombination in active populations mice (TRAP; 5X‐/WT‐TRAP) to permanently label seizure‐activated neurons. We injected AD‐tau unilaterally into the hippocampus and overlying cortex (1 µg/site) at three months of age in 5X‐TRAP and WT‐TRAP littermates. Seizures were then induced with pentylenetetrazol (PTZ) kindling 2‐3 weeks following surgery and seizure‐activated neurons were induced to express tdTomato on the final day of kindling via 4‐hydroxytamoxifen administration. Three months following AD‐tau seeding, 5X‐/WT‐TRAP brains were serially sectioned and underwent immunofluorescent slide scanning. Scanned brain images were then registered to the Allen Brain Atlas and mapped for seizure‐activated neurons (tdTomato+) and tau pathology (phospho‐tau Ser202/Ser205; AT8).

**Results:**

We found that 5X‐TRAP mice had increased tau pathology compared to WT‐TRAP in hippocampal subregions ipsilateral to AD‐tau injection, including the dentate gyrus (p<0.05, n = 5‐10/group) and subiculum (p<0.01, n = 5‐9/group), regardless of seizure kindling, without changes in the contralateral hippocampus. Seizure induction resulted in increased tau spread in remote interconnected brain regions, including the ipsilateral and contralateral thalamo‐cortical regions (p<0.05, n = 5‐9/group). In addition, we found that, compared to neurons from a saline‐treated 5XFAD mouse, thalamic seizure‐activated (tdTomato+) neurons showed significantly increased somatic AT8 levels (p<0.05, n = 8‐11), while surrounding (tdTomato‐) neurons from 5XFAD mice did not, suggesting that these thalamic, seizure‐activated neurons preferentially facilitate tau spread.

**Conclusion:**

Together, our data demonstrate that seizures increase tau spread in mouse models and identify seizures and seizure‐activated neurons as therapeutic targets to slow pathological AD progression.

